# Age and gender specific association between obesity and depressive symptoms: a large-scale cross-sectional study

**DOI:** 10.1186/s12889-020-09664-8

**Published:** 2020-10-17

**Authors:** Wei Liao, Zhicheng Luo, Yitan Hou, Ningning Cui, Xiaotian Liu, Wenqian Huo, Fang Wang, Chongjian Wang

**Affiliations:** 1 Department of Epidemiology and Biostatistics, College of Public Health, Zhengzhou University, Zhengzhou, 100 Kexue Avenue, Zhengzhou, 450001 Henan PR China; 2 Department of Global Health, School of Health Sciences, Wuhan University, Wuhan, Hubei China; 3 Department of Epidemiology, School of Public Health, Shanxi Medical University, Taiyuan, Shanxi PR China

**Keywords:** Depressive symptoms, Obesity, Association, age, gender

## Abstract

**Background:**

This is a cross-sectional study to explore the age and gender specific association between obesity and depression in Chinese rural adults.

**Methods:**

A total of 29,900 eligible participants from the Henan Rural Cohort Study were included. Standard anthropometric measurements were undertaken to obtain data on body mass index (BMI) and waist circumference (WC). The Patient Health Questionnaire-2 (PHQ-2) was utilized to discover depressive symptoms. Logistic regression was performed to explore the association between obesity (independent variable) and depressive symptoms (dependent variable).

**Results:**

There were 1777 subjects with depressive symptoms, accounting for 5.94%. After multivariable adjustment, compared with normal weight group, the odds ratios (ORs) [95% confidence interval (CI)] for depressive symptoms in underweight, overweight and general obese groups were 1.41 (1.08–1.84), 0.87 (0.78–0.97) and 0.86 (0.74–0.99), respectively. Similarly, the OR (95% CI) of abdominal obesity group was 0.84 (0.76–0.93). Besides, there was linear decreasing trend of WC with depressive symptoms, but not BMI. Moreover, the inverse association between obesity and depressive symptoms was stronger in men and the elderly than that in women and the young.

**Conclusion:**

Underweight was associated with a higher prevalence of depressive symptoms, which indicated that health care should pay attention to underweight as well as obesity, especially for women and the young.

**Clinical trial registration:**

The Henan Rural Cohort Study has been registered at Chinese Clinical Trial Register (Registration number: ChiCTR-OOC-15006699). Date of registration: 2015-07-06.

## What is already known on this topic?

Previous studies have investigated the association between obesity and depression, but the association remains controversial. Studies in Western countries have shown that obesity increases the risk of depression, while several Chinese studies have shown that obesity decreases the risk of depression. In addition, there were scarce studies conducted in Chinese rural areas and how gender and age play a role in the association remains unclear.

## What does this study add?

This study found that overweight and obesity associated with a lower prevalence of depressive symptoms in the Chinese rural population. Furthermore, the findings illustrated that the inverse association between obesity and depressive symptoms was stronger in men and the elderly than that in women and the young.

## How might our results change the direction of research or the focus of clinical practice?

Underweight associated with a higher prevalence of depressive symptoms, which indicated that health care should pay attention to underweight as well as obesity, especially in women and the young.

## Background

Obesity is one of the most prevalent chronic condition in the world, and approximate 1.9 billion and 609 million adults worldwide were reported overweight and obese in 2015, respectively, accounting for approximately 39% of the world’s population [1]. According to the location and characteristics of fat distribution, obesity is divided into abdominal obesity and general obesity. A study from 31 provinces in mainland China showed that the prevalence of abdominal obesity in China was 29.1% from 2012 to 2015, which was a major public health challenge [2]. Over the past few years, epidemiological studies have suggested that overweight and obesity were associated with an increased risk of non-communicable diseases, such as stroke [3], cardiovascular disease, type 2 diabetes, osteoarthritis, and some cancers, contributing to a significant health burden globally [4]. In 2008, the World Health Organization listed major depression as the third leading cause of the burden of global disease, and predicted that it will rank first by 2030 [5]. According to the World Health Organization, 5% of the world’s population (more than 264 million people of all ages) suffers from depression [6].

In recent years, the association between obesity and depression have been investigated in some epidemiological studies. However, the results were controversial between western countries and China. Some studies conducted in western countries reported that obesity was associated with increased depression prevalence [7–9]. There were also some systematic review indicating that obesity increase the risks of depression [10, 11]. In addition, other studies described a U-shaped association between obesity and depression (both underweight and obesity were associated with high levels of depression) [12–14]. However, several studies conducted in China demonstrated a negative correlation between obesity and the risk of depression [15–18]. These inconsistencies may be due to different study populations, different perceptions regarding obesity, different body weight and depression criteria, and other factors.

Previous studies have widely examined the association on children, adolescents or adults [19–21]. However, scarce studies were conducted in Chinese rural adults and how gender and age play a role in the association remains unclear. Therefore, in this large population-based cross-sectional study, we aimed to explore the age and gender specific association between obesity and depression in Chinese rural adults. We hypothesize that there exists a reverse association between obesity and depressive symptoms as most Asian studies found, and this association may distinguish in different gender and age subgroups.

## Methods

### Study population

The study population was selected from the baseline survey of Henan Rural Cohort Study, which was conducted from July 2015 to September 2017. The Henan Rural Cohort Study was approved by the Zhengzhou University Life Science Ethics Committee and conducted in accordance with the principles of the Declaration of Helsinki (Code: [2015] MEC (S128)). Before the study commenced, participants were informed of the study’s purpose, health benefits, and potential hazards. Participants were required to provide informed consent and both the researchers and respondents agreed to use the data for scientific research purposes only.

This study used a multistage stratified cluster sampling method to recruit participants in Yuzhou, Suiping, Tongxu, Xinxiang and Yima counties of Henan province in China. Residents aged from 18 to 79 years were invited to participant in this study. We recruit participants through the local medical institutions and Centers for Disease Control and Prevention. Detailed information on the cohort has been described elsewhere [22].

A total of 39,259 were included in this study. For missing the data on depressive symptoms, 9258 participants were excluded. Due to lacking information on BMI or WC, 101 participants were excluded. Finally, 29,900 participants were included in our analysis.

### Data collection and laboratory methods

A structured questionnaire was asked by well-trained research staff according to face-to-face interview. We collected participants’ information on demographic characteristics, lifestyle factors and individual history of chronic diseases. The demographic characteristics included gender, age in years (18–44, 45–54, 55–64, and 65–79), marital status (married/cohabiting, widowed/divorced/separated, and Single), educational level (Elementary school or below, Junior high school, and Senior high school or above), and average monthly income (< 500 RMB, 500- RMB, and ≥ 1000 RMB). Lifestyle factors included smoking, alcohol drinking and physical activity. Smoking was defined as at least one cigarette per day for six sequential or cumulative months. Alcohol consumption was defined as consuming alcohol at least 12 times per year. Physical activity classified as low, moderate, high level according to International Physical Activity Questionnaire (IPAQ) [23]. Chronic diseases included hypertension, hyperlipemia, diabetes mellitus, coronary heart disease (CHD) and stroke. These common chronic diseases were collected through physical examination, laboratory tests, or self-reports.

In accordance with standardized protocols [24], body height and weight of the participants were measured twice with shoes and coats off and the readings were recorded to the nearest 0.1 cm and 0.1 kg, respectively. WC was also measured twice with a standard tape around the waist about 1 cm above the navel and parallel to the ground. The average readings of the two measures were taken for statistical analysis. BMI was calculated as weight (kilogram) divided by height (meter) squared based on the measurement.

### Definition of obesity

In accordance with the Chinese standard of BMI and WC [25]: BMI < 18.5 kg/m^2^, 18.5 ≤ BMI < 24.0 kg/m^2^, 24.0 ≤ BMI < 28.0 kg/m^2^ and BMI ≥ 28.0 kg/m^2^ were for underweight, normal weight, overweight and general obesity, respectively; WC < 90 cm for men and WC < 80 cm for women were classified as normal waist circumference, and WC ≥ 90 cm for men and WC ≥ 80 cm for women were classified as abdominal obesity. In accordance with the World Health Organization standard of BMI and WC [26]: BMI < 18.5 kg/m^2^, 18.5 ≤ BMI < 24.9 kg/m^2^, 25.0 ≤ BMI < 29.9 kg/m^2^ and BMI ≥ 30.0 kg/m^2^ were for underweight, normal weight, overweight and general obesity, respectively; WC < 102 cm for men and WC < 88 cm for women were classified as normal waist circumference, and WC ≥ 102 cm for men and WC ≥ 88 cm for women were classified as abdominal obesity.

### Assessment of depressive symptoms

The Patient Health Questionnaire-2 (PHQ-2) is an abbreviated version of the Patient Health Questionnaire-9 (PHQ-9), which has been widely used for screening depressive symptoms in epidemiological survey. The screening accuracy of the PHQ-2 was satisfactory, with a Patient Health Questionnaire-2 item cutoff of ≥3 [27]. It is consisted of two core items: “little interest or pleasure in doing things” and “feeling down, depressed, or hopeless”. Each item consists of four levels (0 - never; 1 - several days; 2 - more than half the time; and 3 - nearly every day). Thus, the total scores of PHQ-2 is between 0 and 6. In this study, we utilized PHQ-2 scale to identify participants’ depressive symptoms with a cutoff of 3.

### Statistical analysis

Continuous variables were described by mean with standard deviation (SD), while categorical variables were described by frequency with percentages. T test or chi-square test was utilized to compare differences between depressive symptoms group and non-depressive symptoms group.

The association between obesity (independent variable) and depressive symptoms (dependent variable) was examined by binary logistic regression analyses. Model 1 was unadjusted. Model 2 was further adjusted for age and gender. Model 3 was adjusted for age, gender, educational level, marital status, average monthly individual income, current smoking, current alcohol drinking, physical activity, and individual history of chronic diseases (including coronary heart disease, stroke, hypertension, diabetes and dyslipidemia). To identify the dose-response association between BMI and WC and depressive symptoms, restricted cubic spline [28] model was applied where 21 kg/cm^2^ of BMI and 80 cm of WC were as the reference group. Finally, a visual interaction effect was illustrated in order to explore how the effects of BMI and WC on depressive symptoms altered with age.

The figures were produced using the R language software 3.5.2. Statistical analyses were performed by SPSS 21.0 software package (SPSS Institute, Chicago), and all *P* values were two-tailed with a statistical significance level of 0.05.

## Results

Table 1 presents the demographic and socioeconomic characteristics of the study population stratified by depressive symptoms. The mean age of the 29,900 participants was 55.43 (SD: 12.356) years, and 59.21% were women. In this study, a total of 1777 subjects were identified as having depressive symptoms with a prevalence of 5.94%. Compared with non-depressive group, depressive group were older, had lower educational level and average monthly income, more likely to be women and have chronic disease history (all *P* < 0.05). Individuals with depressive symptoms were less likely to be current smokers, current drinkers and married than those without depressive symptoms (all *P* < 0.05). Besides, individuals with depressive symptoms were more prone to have a lower level of BMI (24.43 vs. 24.75) and WC (82.41 vs. 83.78). Supplementary Table 1 shows the distributions of selected variables of the participants stratified by obesity status. There are significant differences statistically in all demographic and socioeconomic characteristics (all *P* < 0.05).

**Table 1:** Distributions of selected variables of the participants stratified by depressive symptoms status

Variables	Overall (***n*** = 29,900)	Non-DS (***n*** = 28,123)	DS (***n*** = 1777)	***P*** value ^*^
Age (year, mean ± SD)	55.43 ± 12.356	55.38 ± 12.383	56.23 ± 11.894	0.005
Women (n, %)	17,704 (59.21)	16,504 (58.69)	1200 (67.53)	< 0.001
Educational level (n, %)				
Elementary school or below	13,324 (44.23)	12,279 (43.66)	945 (53.18)	< 0.001
Junior high school	11,647 (38.95)	11,022 (39.19)	625 (35.17)	
Senior high school or above	5029 (16.82)	4822 (17.15)	207 (11.65)	
Marital status (n, %)				
Married/cohabiting	26,968 (90.19)	25,407 (90.34)	1561 (87.84)	0.002
Widowed/separated/divorced	2423 (8.10)	2242 (7.97)	181 (10.19)	
Single	509 (1.71)	474 (1.69)	35 (1.97)	
Average monthly income (n, %)				
< 500 RMB	10,795 (36.10)	9954 (35.39)	841 (47.32)	< 0.001
500- RMB	9416 (31.49)	8948 (31.82)	468 (26.34)	
≥ 1000 RMB	9689 (32.41)	9221 (32.79)	468 (26.34)	
Physical activity (n, %)				
Low	9501 (31.78)	8905 (31.66)	596 (33.54)	0.177
Moderate	11,009 (36.82)	10,357 (36.83)	652 (36.69)	
High	9390 (31.40)	8861 (31.51)	529 (29.77)	
Current smokers (n, %)	6005 (20.08)	5725 (20.36)	280 (15.76)	< 0.001
Current drinkers (n, %)	5219 (17.45)	5008 (17.81)	211 (11.87)	< 0.001
Chronic disease (n, %)	17,755 (59.38)	16,654 (59.50)	1101 (62.06)	0.033
Body mass index (kg/m^2^, mean ± SD)	24.73 ± 3.569	24.75 (3.559)	24.43 (3.708)	< 0.001
Waist circumference (cm, mean ± SD)	83.70 ± 10.469	83.78 (10.450)	82.41 (10.699)	< 0.001

Abbreviation: *DS* depressive symptoms; *SD* standard deviation; *RMB* Renminbi

^*^ T-test was performed to compare the differences in continuous variables; Chi-square test was used to compare the differences in the categorical variables

**Fig. 1:**
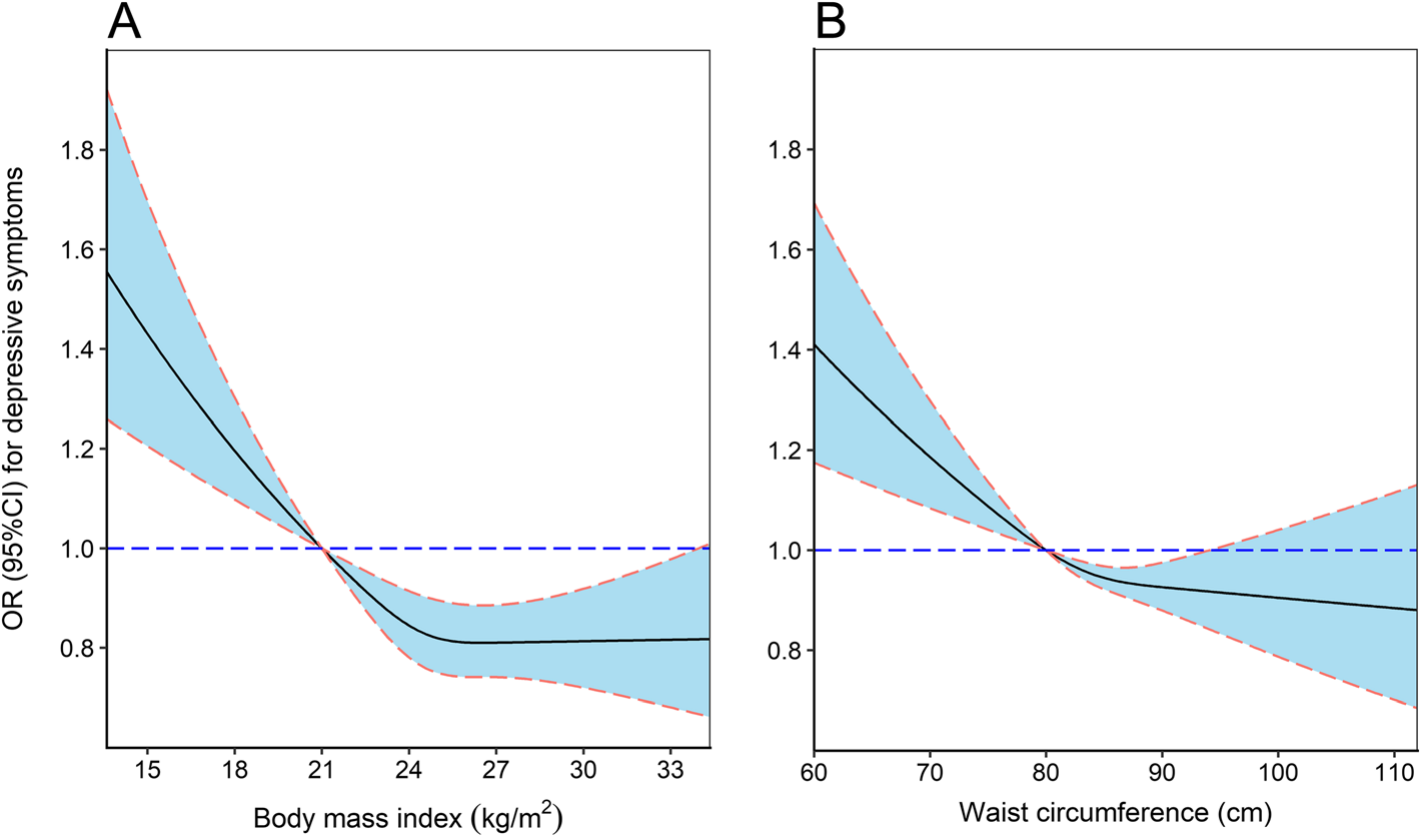
The dose-response relationships of BMI and WC with depressive symptoms

Odds ratios for depressive symptoms associated with BMI and WC are presented in Table 2. BMI and WC were considered as both categorical variables according to Chinese criteria and continuous variable scaled to 1-kg/m^2^ and 1-cm increase, respectively. After multivariable adjustment, compared with normal weight group, the odds ratios for depressive symptoms in underweight, overweight and general obese groups were 1.41 (1.08–1.84), 0.87 (0.78–0.97) and 0.86 (0.74–0.99), respectively. Moreover, the OR of depressive symptoms associated with 1 kg/m^2^ increase in BMI was 0.97 (0.95–0.98). Similarly, abdominal obesity was associated with a lower prevalence of depressive symptoms. Supplementary Table 2 presents the association between obesity and depressive symptoms according to BMI and WC in WHO definition. The results were consistent with the results using Chinese standards. The dose-response relationships of BMI and WC with depressive symptoms are further evaluated through the restricted cubic spline curves in Fig. 1, which suggested that the prevalence of depressive symptoms may be lower with a higher level of WC (*P* for non-linear trend =0.108), but not BMI (*P* for non-linear trend = 0.0170).

**Table 2:** Association between obesity and depressive symptoms according to BMI and WC

Variables	No. of cases	Model 1	Model 2	Model 3
		OR (95% CI)	OR (95% CI)	OR (95% CI)
**BMI (kg/m**^**2**^**)**				
**Continuous**	29,900	0.97 (0.96–0.99)	0.97 (0.96–0.99)	0.97 (0.95–0.98)
**Category**				
Underweight (< 18.5)	779	1.44 (1.11–1.87)	1.43 (1.11–1.86)	1.41 (1.08–1.84)
Normal weight (18.5–23.9)	12,365	Reference	Reference	Reference
Overweight (24.0–27.9)	11,668	0.89 (0.80–0.99)	0.89 (0.80–0.99)	0.87 (0.78–0.97)
General obesity (≥28.0)	5088	0.90 (0.78–1.04)	0.88 (0.77–1.02)	0.86 (0.74–0.99)
*P* for trend		0.003	0.002	0.001
**WC (cm)**				
**Continuous**	29,900	0.99 (0.98–0.99)	0.99 (0.98–0.99)	0.99 (0.98–0.99)
**Category** ^**a**^				
Normal WC	15,143	Reference	Reference	Reference
Abdominal obesity	14,757	0.95 (0.86–1.04)	0.85 (0.77–0.93)	0.84 (0.76–0.93)

Abbreviation: *BMI* body mass index; *WC* waist circumference

Model 1: unadjusted;

Model 2: adjusted for age and gender;

Model 3: adjusted for age, gender, educational level, marital status, average monthly income, physical activity, current smoking, current drinking and chronic disease (including coronary heart disease, stroke, hypertension, diabetes and dyslipidemia)

^a^ Abdominal obesity was classified as WC ≥ 80 cm for women and WC ≥ 90 cm for men

Table 3 demonstrates the association between BMI and WC and depressive symptoms according to gender. Among men, the risks of depressive symptoms in underweight, overweight and general obese groups were 1.62 (1.08–2.43), 0.83 (0.69–0.99) and 0.68 (0.52–0.90) compared with normal weight group in the crude model. After multivariable adjustment, the ORs in underweight and overweight groups became borderline non-significant. However, there were no significant associations between BMI groups and depressive symptoms among women. According to the WC category, compared with those who had normal waist circumference, participants with abdominal obesity associated with a lower prevalence of depressive symptoms among men and women. Notably, the inverse association between abdominal obesity and depressive symptoms was stronger in men than that in women (*P* < 0.05).

**Table 3:** Gender-specific association between obesity and depressive symptoms

Variables	No. of cases	Model 1	Model 2	Model 3
		OR (95% CI)	OR (95% CI)	OR (95% CI)
**BMI (kg/m**^**2**^**)**				
**Men**				
Underweight	342	1.62 (1.08–2.43)	1.64 (1.09–2.45)	1.47 (0.98–2.23)
Normal weight	5339	Reference	Reference	Reference
Overweight	4640	0.83 (0.69–0.99)	0.82 (0.68–0.99)	0.85 (0.70–1.02)
General obesity	1875	0.68 (0.52–0.90)	0.67 (0.51–0.88)	0.67 (0.51–0.90)
*P* for trend		< 0.001	< 0.001	0.001
**Women**				
Underweight	437	1.34 (0.96–1.88)	1.33 (0.95–1.86)	1.36 (0.97–1.92)
Normal weight	7026	Reference	Reference	Reference
Overweight	7028	0.92 (0.80–1.04)	0.90 (0.79–1.03)	0.88 (0.77–1.01)
General obesity	3213	0.98 (0.83–1.15)	0.96 (0.81–1.13)	0.91 (0.77–1.08)
*P* for trend		0.194	0.127	0.045
***P*** **for interaction**		0.015	0.021	0.136
**WC (cm)**^**#**^				
**Men**				
Normal WC	8037	Reference	Reference	Reference
Abdominal obesity	4159	0.73 (0.61–0.88)	0.73 (0.60–0.87)	0.76 (0.62–0.92)
**Women**				
Normal WC	7106	Reference	Reference	Reference
Abdominal obesity	10,598	0.92 (0.82–1.03)	0.88 (0.78–0.99)	0.85 (0.76–0.97)
***P*** **for interaction**		0.043	0.068	0.426

Abbreviation: *BMI* body mass index; *WC* waist circumference

Model 1: unadjusted;

Model 2: adjusted for age and gender;

Model 3: adjusted for age, gender, educational level, marital status, average monthly income, physical activity, current smoking, current drinking and chronic disease (including coronary heart disease, stroke, hypertension, diabetes and dyslipidemia)

^#^ Abdominal obesity was classified as WC ≥ 80 cm for women and WC ≥ 90 cm for men

**Fig. 2:**
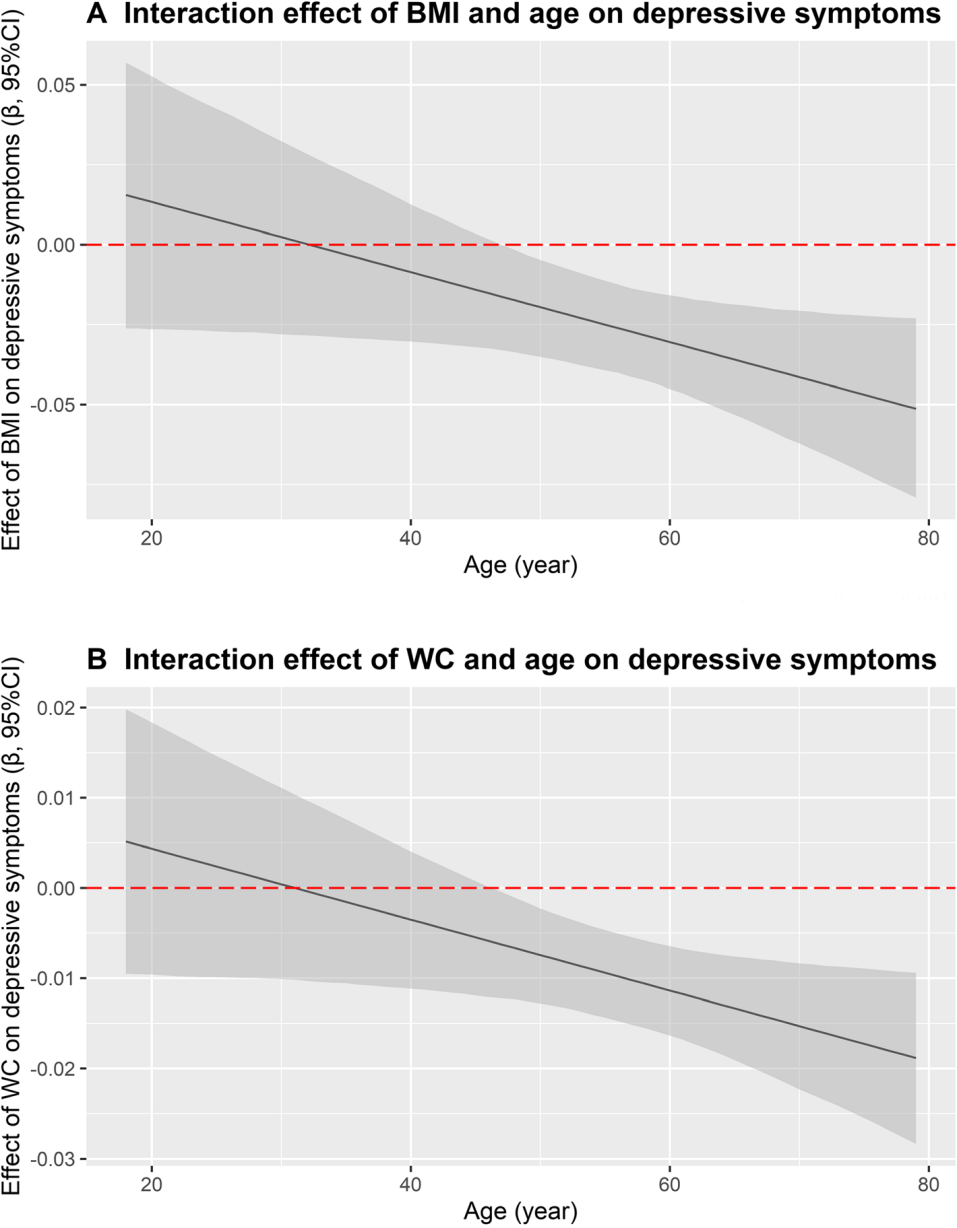
Interactive association of BMI/WC and age on depressive symptoms

Age specific association between obesity and depressive symptoms is shown in Table 4. Among participants aged from 18 to 44 year, the risks of depressive symptoms in underweight, overweight and general obese groups were all higher than that in normal weight group, although these associations were not statistically significant. In the other two age groups, underweight participants had higher ORs for depressive symptoms while participants with overweight and general obesity had lower ORs compared with normal weight people. According to the WC category, participants with abdominal obesity associated with a lower prevalence of depressive symptoms than those with normal waist circumference among people aged 45 years or above. Gender and age specific association between obesity and depressive symptoms according to WHO criteria are listed in the Supplementary Table 2. The results were consistent with the results using Chinese standards. In addition, interactive association of BMI/WC and age on depressive symptoms is illustrated in Fig. 2. As shown, the negative associations of BMI/WC with depressive symptoms were enhanced by increasing age (both *P* < 0.05). The interactive association of BMI/WC and age on depressive symptoms in men and women are shown in Supplementary Fig. 1 and 2, respectively. The results demonstrated that the negative associations of BMI/WC with depressive symptoms were enhanced by increasing age in both men and women.

**Table 4:** Age-specific association between obesity and depressive symptoms

Variables	No. of cases	Model 1	Model 2	Model 3
		OR (95% CI)	OR (95% CI)	OR (95% CI)
**BMI (kg/m**^**2**^**)**				
**18–44 years old**				
Underweight	53	1.48 (0.80–2.74)	1.48 (0.80–2.74)	1.47 (0.78–2.74)
Normal weight	733	Reference	Reference	Reference
Overweight	773	1.09 (0.83–1.44)	1.09 (0.83–1.44)	1.10 (0.83–1.46)
General obesity	446	1.23 (0.88–1.71)	1.23 (0.88–1.71)	1.24 (0.87–1.76)
*P* for trend		0.465	0.481	0.468
**45–59 years old**				
Underweight	49	1.81 (1.06–3.09)	1.77 (1.03–3.01)	1.76 (1.03–3.03)
Normal weight	1733	Reference	Reference	Reference
Overweight	1837	0.88 (0.74–1.05)	0.87 (0.73–1.03)	0.85 (0.72–1.02)
General obesity	810	0.86 (0.69–1.07)	0.85 (0.68–1.06)	0.81 (0.64–1.01)
*P* for trend		0.032	0.022	0.010
**60–79 years old**				
Underweight	240	1.32 (0.94–1.86)	1.33 (0.94–1.87)	1.26 (0.89–1.79)
Normal weight	2873	Reference	Reference	Reference
Overweight	2030	0.84 (0.71–0.98)	0.80 (0.68–0.94)	0.81 (0.68–0.95)
General obesity	619	0.83 (0.67–1.04)	0.76 (0.61–0.94)	0.75 (0.60–0.94)
*P* for trend		0.004	< 0.001	0.001
***P*** **for interaction**		0.047	0.006	0.021
**WC (cm)**^**#**^				
**18–44 years old**				
Normal WC	1218	Reference	Reference	Reference
Abdominal obesity	787	0.94 (0.74–1.21)	0.94 (0.74–1.21)	0.96 (0.74–1.25)
**45–59 years old**				
Normal WC	2685	Reference	Reference	Reference
Abdominal obesity	1744	0.96(0.82–1.11)	0.86 (0.73–1.01)	0.84 (0.71–0.99)
**60–79 years old**				
Normal WC	4134	Reference	Reference	Reference
Abdominal obesity	1628	0.93 (0.81–1.08)	0.76 (0.65–0.89)	0.77 (0.65–0.90)
***P*** **for interaction**		0.238	0.035	0.087

Abbreviation: *BMI* body mass index; *WC* waist circumference

Model 1: unadjusted;

Model 2: adjusted for age and gender;

Model 3: adjusted for age, gender, educational level, marital status, average monthly income, physical activity, current smoking, current drinking and chronic disease (including coronary heart disease, stroke, hypertension, diabetes and dyslipidemia)

^#^ Abdominal obesity was classified as WC ≥ 80 cm for women and WC ≥ 90 cm for men

## Discussion

To the best of our knowledge, this research is the first study to explore the association of obesity with depression in Chinese rural population. The inverse association between obesity and depression were found in this study. Besides, there was linear trend for the dose-response relationships of WC with depressive symptoms, but not BMI. In addition, the inverse association between abdominal obesity and depressive symptoms was stronger in men than that in women. The inverse association between BMI/WC and depressive symptoms increased as age increased.

Our finding of the inverse association between obesity and depression among Chinese rural population was consistent with those reported in Korea, Taiwan and Japan [19, 29–31]. In addition, some previous studies in China have also observed an inverse association between obesity and depression [15, 16]. Conversely, some studies conducted in western countries reported that obesity was associated with increased depression prevalence [32–34]. These inconsistencies may be due to different study populations, different perceptions regarding obesity, different body weight and depression criteria, and other factors.

The negative relationship between obesity and depression can be explained by the “jolly fat” hypothesis which was first reported by Crisp and his colleagues [35]. Crisp proposed that there was a significant positive relationship between substantial obesity and low levels of depression in men. The special phenomenon has also been found in several other studies [36–38]. This hypothesis could be explained by losing weight through diet restriction which may be an important factor in inducing depression. Thus, people who lost weight through dieting have high risk of depression [35].

In this study, the participants are Chinese rural population; therefore, Chinese traditional culture may be another factor influencing the inverse relationship between obesity and depression. In Chinese traditional culture, having a good appetite is a blessing and being obesity represents good social and economic status [39]. It is different from western countries that excessive body weight was usually stigmatized [40].

The inverse relationship may also be the result of biological molecular mechanism. Neuropeptide Y (NPY) is a 36-amino acid peptide that widely distributed in the central nervous system and it may explain the association between increased body mass and reduced depressive symptoms [41]. According to the result of forced swimming test on mice which was widely used to screen for potential antidepressants [42], NPY treatment could increase the number of swimming times of animals and reduce the immobilization of the forced swimming test, indicating that NPY had antidepressant effect and could increase appetite [43].

The current study indicated that the inverse association between abdominal obesity and depressive symptoms was stronger in men than that in women. It may be due to women had more hormonal fluctuations and excessive sensitivity to hormonal fluctuations than men [44]. Besides, there were also a lot of other factors accounting for the increased probability of depression among women such as psychosocial events, victimization, sex-specific socialization, internalization coping style, and disadvantaged social status [45].

Furthermore, the inverse association of BMI and WC with depressive symptoms became stronger as age increased. It may result from the difference on the self-perception of being fat between the young and old. Having a self-perception of being fat produced a potentiating effect, significantly increasing the likelihood of depression [46]. In addition, nowadays, images of unrealistically thin and stigmatization of obesity are disseminated in the current society [47, 48]. Therefore, the internalization of the media ideal of thinness affects more and more modern people, especially the young ones.

The large sample size is an advantage of this study. There were some limitations in our study. First, we could not identify the causal relationships between obesity and depression because the study was conducted at the cross-sectional level. Thus, more prospective studies need to be done. Second, depressive symptoms in our study were assessed by the Patient Health Questionnaire-2 (PHQ-2) based on self-report of participants, which might cause recall bias. Although our scale is short, it is effective and very applicable in the large epidemiologic field investigation. In addition, as an important factor for both obesity and depressive symptoms, diet was not included in the analysis which may affect our results.

## Conclusions

The results of this study suggested that obesity was associated with a lower prevalence of depressive symptoms, which supported the “jolly fat” hypothesis in China. The findings indicated that health care should pay attention to underweight as well as obesity. In addition, gender and age specific differences between obesity measures and depressive symptoms were found. Thus, targeted strategies on preventing depression are needed to pay more attention to women and the young. In the future, prospective studies are wanted to better explore the mechanism of this association.

## Supplementary information

**Additional file 1.**

## Data Availability

The data analyzed during current study are available from the corresponding author on reasonable request.

## Abbreviations

BMI: Body mass index
CI: Confidence interval
CHD: Coronary heart disease
DS: Depressive symptoms
IPAQ: International Physical Activity Questionnaire
NPY: Neuropeptide Y
ORs: Odds ratios
PHQ-2: Patient Health Questionnaire-2
PHQ-9: Patient Health Questionnaire-9
RMB: Renminbi
SD: Standard deviation
WC: Waist circumference
WHO: World Health Organization
