# Supplementary material for: Age and gender specific association between obesity and depressive symptoms: a large-scale cross-sectional study
Source: BMC Public Health. 2020 Oct 17;20:1565. doi: 10.1186/s12889-020-09664-8 (PMC7568408; doi:10.1186/s12889-020-09664-8)
Supplement: Supplementary file 1 — Additional file 1. [file 12889_2020_9664_MOESM1_ESM.docx]

**Supplementary table 1.** **Distributions of selected variables of the participants stratified by obesity status.**

| **Variables** |  | **BMI** | | | | ***P* value^*^** |  | **WC** | | ***P* value^*^** |
| --- | --- | --- | --- | --- | --- | --- | --- | --- | --- | --- |
|  |  | **Underweight** | **Normal weight** | **Overweight** | **General obesity** |  |  | **Normal WC** | **Abdominal obesity** |  |
| Age (year, mean ± SD) |  | 57.03 ± 16.351 | 55.59 ± 13.088 | 55.47 ± 11.502 | 54.72 ± 11.648 | <0.001 |  | 54.92 ± 13.212 | 55.95 ± 11.387 | <0.001 |
| Women (n, %) |  | 437(56.10) | 7026(56.82) | 7028(60.23) | 3213(63.15) | <0.001 |  | 7106(46.93) | 10598(71.82) | <0.001 |
| Educational level (n, %) |  |  |  |  |  | 0.003 |  |  |  | <0.001 |
| Elementary school or below |  | 384(49.29) | 5412(43.77) | 5091(43.63) | 2337(45.93) |  |  | 6240(41.20) | 6984(47.33) |  |
| Junior high school |  | 261(33.50) | 4883(39.49) | 4594(39.37) | 1909(37.52) |  |  | 6108(40.34) | 5539(37.53) |  |
| Senior high school or above |  | 134(17.21) | 2070(16.74) | 1983(17.00) | 842(16.55) |  |  | 2795(18.46) | 2234(15.14) |  |
| Marital status (n, %) |  |  |  |  |  | <0.001 |  |  |  | <0.001 |
| Married/cohabiting |  | 628(80.62) | 10990(88.88) | 10670(91.44) | 4680(91.98) |  |  | 13500(89.15) | 13468(91.27) |  |
| Widowed/separated/divorced |  | 93(11.94) | 1079(8.73) | 891(7.64) | 360(7.08) |  |  | 1217(8.04) | 1206(8.17) |  |
| Single |  | 58(7.44) | 296(2.39) | 107(0.92) | 48(0.94) |  |  | 426(2.81) | 83(0.56) |  |
| Average monthly income (n, %) |  |  |  |  |  | <0.001 |  |  |  | <0.001 |
| <500 RMB |  | 325(41.72) | 4698(37.99) | 4093(35.08) | 1679(33.00) |  |  | 5673(37.46) | 5122(34.71) |  |
| 500-1000 RMB |  | 232(29.78) | 3708(29.99) | 3790(32.48) | 1686(33.14) |  |  | 4614(30.47) | 4802(32.54) |  |
| ≥1000 RMB |  | 222(28.50) | 3959(32.02) | 3785(32.44) | 1723(33.86) |  |  | 4856(32.07) | 4833(32.75) |  |
| Physical activity (n, %) |  |  |  |  |  | <0.001 |  |  |  | <0.001 |
| Low |  | 255(32.73) | 3696(29.89) | 3673(31.48) | 1877(36.89) |  |  | 4483(29.60) | 5018(34.00) |  |
| Moderate |  | 323(41.46) | 4674(37.80) | 4294(36.80) | 1718(33.77) |  |  | 5505(36.35) | 5504(37.30) |  |
| High |  | 201(25.81) | 3995(32.31) | 3701(31.72) | 1493(29.34) |  |  | 5155(34.05) | 4235(28.70) |  |
| Current smokers (n, %) |  | 174(22.34) | 2865(23.17) | 2149(18.42) | 817(16.06) | <0.001 |  | 4082(26.96) | 1923(13.03) | <0.001 |
| Current drinkers (n, %) |  | 84(10.78) | 2021(16.34) | 2131(18.26) | 983(19.32) | <0.001 |  | 3058(20.19) | 2161(14.64) | <0.001 |
| Chronic disease (n, %) |  | 243(31.27) | 5780(46.97) | 7722(66.45) | 4010(79.25) | <0.001 |  | 7124(47.24) | 10631(72.40) | <0.001 |
| PHQ-2 score |  | 0.74 ± 1.372 | 0.54 ± 1.154 | 0.49 ± 1.100 | 0.47 ± 1.086 | <0.001 |  | 0.53 ± 1.154 | 0.49 ± 1.102 | 0.001 |

Abbreviation: BMI: body mass index; WC: waist circumference; SD, standard deviation; RMB, Renminbi; PHQ-2, Patient Health Questionnaire-2.

Chronic disease: including coronary heart disease, stroke, hypertension, diabetes and dyslipidemia. Whoever suffering one of the above diseases was defined as having chronic disease.

^*^ Analysis of variance was performed to compare the differences in continuous variables; Chi-square test was used to compare the differences in the categorical variables.

**Supplementary table 2.** **Association between obesity and depressive symptoms according to BMI and WC in WHO definition.**

| **Variables** | **No. of cases** |  | **Model 1** |  | **Model 2** |  | **Model 3** |
| --- | --- | --- | --- | --- | --- | --- | --- |
|  |  |  | OR (95% CI) |  | OR (95% CI) |  | OR (95% CI) |
| **BMI (kg/m^2^)** |  |  |  |  |  |  |  |
| Underweight (<18.5) | 779 |  | 1.45 (1.12-1.87) |  | 1.45 (1.12-1.88) |  | 1.42 (1.10-1.85) |
| Normal weight (18.5-24.9) | 15689 |  | Reference |  | Reference |  | Reference |
| Overweight (25.0-29.9) | 11218 |  | 0.88 (0.79-0.97) |  | 0.87 (0.78-0.96) |  | 0.85 (0.77-0.95) |
| General obesity (≥30.0) | 2214 |  | 0.88 (0.73-1.07) |  | 0.86 (0.71-1.04) |  | 0.82 (0.67-1.00) |
| *P* for trend |  |  | 0.001 |  | <0.001 |  | <0.001 |
| **WC (cm) ^#^** |  |  |  |  |  |  |  |
| Normal WC | 23874 |  | Reference |  | Reference |  | Reference |
| Abdominal obesity | 6026 |  | 0.99 (0.87-1.11) |  | 0.84 (0.74-0.96) |  | 0.82 (0.72-0.93) |

Abbreviation: BMI: body mass index; WC: waist circumference.

Model 1: unadjusted;

Model 2: adjusted for age and gender;

Model 3: adjusted for age, gender, educational level, marital status, average monthly income, physical activity, current smoking, current drinking and chronic disease (including coronary heart disease, stroke, hypertension, diabetes and dyslipidemia).

^#^ Abdominal obesity was classified as WC ≥ 88 cm for women and WC ≥ 102 cm for men according to the WHO criteria.

**Supplementary table 3.** **Gender and age specific** **association between obesity and depressive symptoms according to WHO criteria.**

| **Variables** | **Men** | **Women** |  | **18-44 years old** | **45-59 years old** | **60-79 years old** |
| --- | --- | --- | --- | --- | --- | --- |
|  | OR (95% CI) | OR (95% CI) |  | OR (95% CI) | OR (95% CI) | OR (95% CI) |
| **BMI (kg/m^2^)** |  |  |  |  |  |  |
| Underweight | 1.47 (0.97-2.21) | 1.40 (1.00-1.97) |  | 1.44 (0.77-2.69) | 1.77 (1.03-3.03) | 1.30 (0.92-1.83) |
| Normal weight | Reference | Reference |  | Reference | Reference | Reference |
| Overweight | 0.73 (0.60-0.89) | 0.90 (0.79-1.02) |  | 1.12 (0.85-1.48) | 0.80 (0.68-0.95) | 0.80 (0.68-0.95) |
| General obesity | 0.72 (0.49-1.07) | 0.84 (0.67-1.06) |  | 1.12 (0.71-1.76) | 0.80 (0.59-1.08) | 0.73 (0.53-1.00) |
| ***P* for trend** | <0.001 | 0.010 |  | 0.745 | 0.003 | 0.001 |
| ***P* for interaction** | 0.136 | |  | 0.021 | | |
| **WC (cm)** ^#^ |  |  |  |  |  |  |
| Normal WC | Reference | Reference |  | Reference | Reference | Reference |
| Abdominal obesity | 0.73 (0.49-1.10) | 0.82 (0.71-0.93) |  | 0.95 (0.66-1.37) | 0.88 (0.72-1.07) | 0.72 (0.59-0.87) |
| ***P* for interaction** | 0.426 | |  | 0.087 | | |

Abbreviation: BMI: body mass index; WC: waist circumference.

The model adjusted for age, gender, educational level, marital status, average monthly income, physical activity, current smoking, current drinking and chronic disease (including coronary heart disease, stroke, hypertension, diabetes and dyslipidemia).

^#^ Abdominal obesity was classified as WC ≥ 88 cm for women and WC ≥ 102 cm for men according to the WHO criteria.


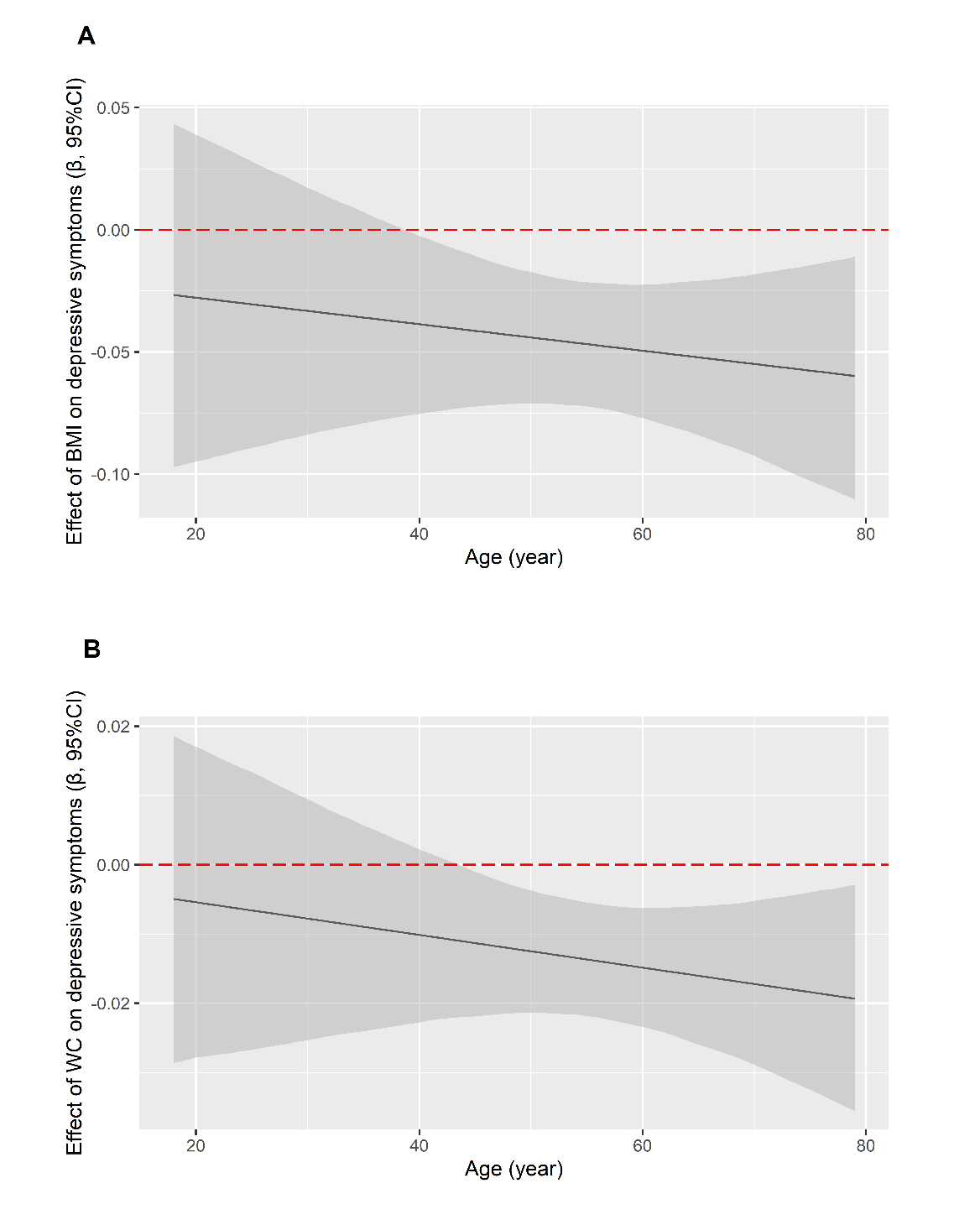
Supplementary figure 1. Interactive association of BMI/WC and age on depressive symptoms in men (A: interactive association of BMI and age on depressive symptoms; B: interactive association of WC and age depressive symptoms).


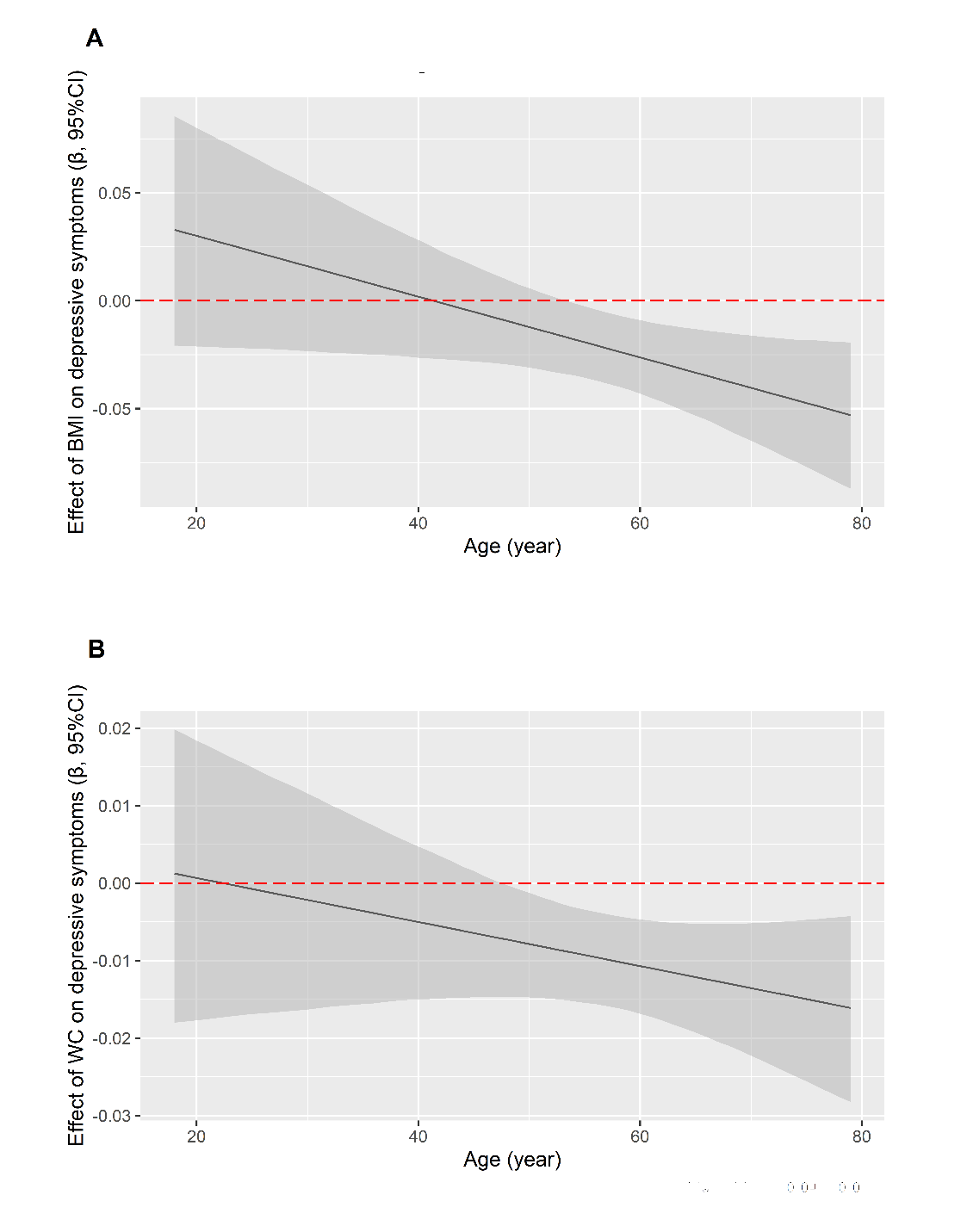
Supplementary figure 2. Interactive association of BMI/WC and age on depressive symptoms in women (A: interactive association of BMI and age on depressive symptoms; B: interactive association of WC and age depressive symptoms).
